# Molecular insights into mineralotropic hormone inter-regulation

**DOI:** 10.3389/fendo.2023.1213361

**Published:** 2023-06-27

**Authors:** J. Wesley Pike, Seong Min Lee, Mark B. Meyer

**Affiliations:** ^1^ Department of Biochemistry, University of Wisconsin-Madison, Madison, WI, United States; ^2^ Department of Nutritional Sciences, University of Wisconsin-Madison, Madison, WI, United States

**Keywords:** mineral regulating hormones, transcription, ChIP-seq analysis, CRISPR/Cas9, mutant mice, Cyp27b1/Cyp24a1 genes, PTH gene, FGF23 gene

## Abstract

The regulation of mineral homeostasis involves the three mineralotropic hormones PTH, FGF23 and 1,25-dihydroxyvitamin D_3_ (1,25(OH)_2_D_3_). Early research efforts focused on PTH and 1,25(OH)_2_D_3_ and more recently on FGF23 have revealed that each of these hormones regulates the expression of the other two. Despite early suggestions of transcriptional processes, it has been only recently that research effort have begun to delineate the genomic mechanisms underpinning this regulation for 1,25(OH)_2_D_3_ and FGF23; the regulation of PTH by 1,25(OH)_2_D_3_, however, remains obscure. We review here our molecular understanding of how PTH induces *Cyp27b1* expression, the gene encoding the enzyme responsible for the synthesis of 1,25(OH)_2_D_3_. FGF23 and 1,25(OH)_2_D_3_, on the other hand, function by suppressing production of 1,25(OH)_2_D_3_. PTH stimulates the PKA-induced recruitment of CREB and its coactivator CBP at CREB occupied sites within the kidney-specific regulatory regions of *Cyp27b1*. PKA activation also promotes the nuclear translocation of SIK bound coactivators such as CRTC2, where it similarly interacts with CREB occupied *Cyp27b1* sites. The negative actions of both FGF23 and 1,25(OH)_2_D_3_ appear to suppress *Cyp27b1* expression by opposing the recruitment of CREB coactivators at this gene. Reciprocal gene actions are seen at *Cyp24a1*, the gene encoding the enzyme that degrades 1,25(OH)_2_D_3_, thereby contributing to the overall regulation of blood levels of 1,25(OH)_2_D_3_. Relative to PTH regulation, we summarize what is known of how 1,25(OH)_2_D_3_ regulates PTH suppression. These studies suggest that it is not 1,25(OH)_2_D_3_ that controls PTH levels in healthy subjects, but rather calcium itself. Finally, we describe current progress using an *in vivo* approach that furthers our understanding of the regulation of *Fgf23* expression by PTH and 1,25(OH)_2_D_3_ and provide the first evidence that P may act to induce *Fgf23* expression via a complex transcriptional mechanism in bone. It is clear, however, that additional advances will need to be made to further our understanding of the inter-regulation of each of these hormonal genes.

## Introduction

**Figure 1:**
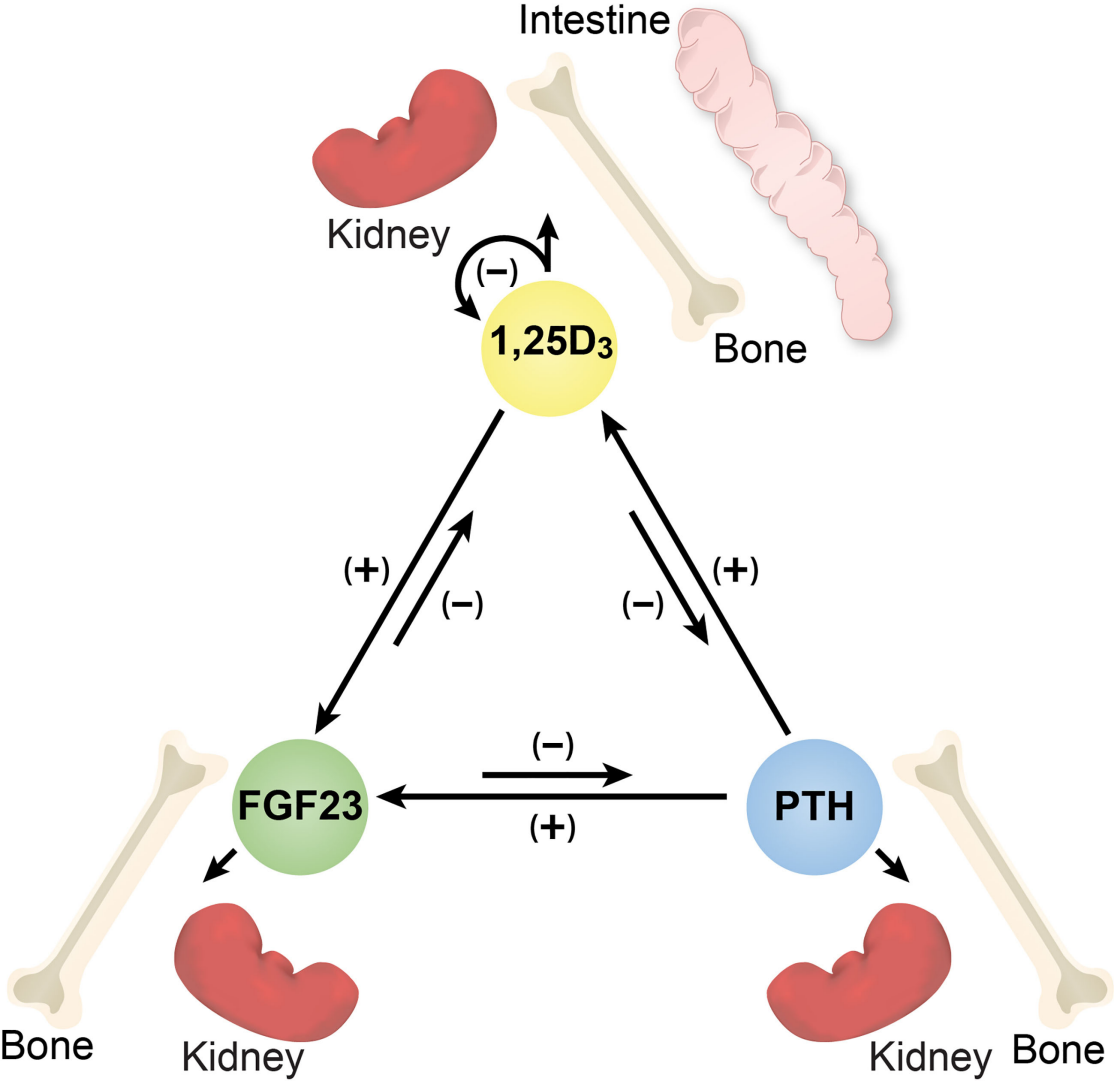
Diagram of the inter-regulation of PTH, FGF23 and 1,25(OH)_2_D_3_ and the target tissues relevant to each hormone. Arrows together with (+) or (-) signs indicate the direction and influence of each hormone.

The maintenance of mineral metabolism is regulated predominantly by 1,25-dihydroxyvitamin D_3_ (1,25(OH)_2_D_3_), PTH and FGF23 (1, 2). While the effective concentrations of each of these hormones in the circulation is regulated through multiple mechanisms, all three are fundamentally derived in a tissue-specific manner via transcription from their respective genes. Interestingly, while early physiological observations led to the realization that each of these hormones also contributed significantly to the abundance of the other two, it has been only recently that research efforts have begun to identify the underlying molecular mechanisms responsible for altering the expression of these hormonal genes (2). The consequences of this inter-regulation, although frequently impacted by downstream non-transcriptional events as well, is the integration of the activities of these three different hormones with sometimes overlapping and often inhibitory actions at certain mineral regulating tissues including kidney and bone (**Figure 1**). There are, of course, frequent novel actions of each of these hormones at numerous tissues that are not involved in the active regulation of mineral homeostasis that further complicate the picture. In this paper, we consider advances that we have made at the genomic level that underlie the production of 1,25(OH)_2_D_3_ by PTH, FGF23 and 1,25(OH)_2_D_3_ through their actions on the *Cyp27b1* and *Cyp24a1* genes expressed in renal proximal tubules. We also review existing support for the mechanisms through which 1,25(OH)_2_D_3_ has been proposed in early studies to regulate PTH transcription, although advances here are limited. Finally, we discuss the results of our recent studies of the regulation of *Fgf23* transcription using an approach involving gene modification via CRISPR-Cas9 gene editing followed by loss of *Fgf23* regulation studies *in vivo*. These efforts attempt to identify the genomic control regions within the *Fgf23* gene locus that mediate the regulatory actions of 1,25(OH)_2_D_3_, PTH and phosphate (P) at this gene and ultimately to identify unique factors involved in these transcriptional mechanisms. While our focus is on hormonal inter-regulation, it is worth noting that calcium (Ca) and P do not appear to directly control the expression of the renal *Cyp27b1 and Cyp24a1* genes, but rather that Ca directly regulates PTH expression whereas P controls FGF23 levels; these in turn then control the production of 1,25(OH)_2_D_3_. For this reason, we also include a brief discussion of Ca and a direct consideration of P, with a particular emphasis on P regulation of *Fgf23* expression. It will also be evident that it has been the application of several new technologies that helped advance our understanding of this inter-regulation, particularly that regarding regulation of PTH and FGF23. This paper does not represent a thorough review of these subjects, but rather a summary of the recent work that we have done aimed at understanding the molecular mechanisms of this regulation.

## New insights into transcriptional activation of genes

Before we address the regulation of renal 1,25(OH)_2_D_3_ which represents the primary endocrine source of this hormone, followed by parathyroid gland-produced PTH and bone-derived FGF23, we begin this paper by discussing the new approaches that we have taken to identify the mechanisms involved in gene regulation. Indeed, the development of chromatin-immunoprecipitation (ChIP) and other genomic procedures coupled to next-generation DNA sequencing technics (ChIP-seq analysis) and their applications have fully changed the way in which gene regulation is explored (3, 4). ChIP-seq analysis has seen widespread application in part to identify and characterize ligand-induced transcription factor DNA binding activity, to examine the recruitment of coregulators to these DNA bound factors at specific genomic sites, and to assess actions downstream of this binding, such as the recruitment of additional protein participants in the transcriptional pathways such as RNA polymerase II in an unbiased, genome-wide fashion. These studies have revealed a multitude of new details inherent to transcriptional processes at single genes and on a genome-wide scale; some of these principles confirm what has been previously determined while others identify modifications to those already known to exist. Others have advanced our understanding, revealing entirely new principles of gene regulation that must be now considered in mechanistic studies. Perhaps as important, they have also revealed how inherent technical bias drawn from over three decades of analyses using *in vitro* molecular biological methods involving cell-based transfection studies have led to numerous faulty conclusions regarding transcriptional mechanisms.

Regarding the molecular actions of vitamin D, genome-wide studies using ChIP-seq analysis, as summarized in **Table 1**, have revealed many findings that are relevant not only to vitamin D actions but to the general actions of transcription factors that mediate the activities of numerous hormones and other regulatory components. As can be seen, these analyses in both human and mouse cultured cells as well as tissues *in vivo* indicate several thousand binding sites for the vitamin D receptor (VDR), a number consistent with other regulatory factors; the number and location of these sites for transcription factors in general are, however, dependent upon the factor involved, and are affected by species, the type of target cell studied, and nutritional status, among other variables (5). In the case of the VDR, most of these sites, but not all, are highly dependent upon 1,25(OH)_2_D_3_ pretreatment, indicating that like many nuclear receptors (NRs), the VDR represents a ligand-dependent DNA binding factor (6). The regulation of DNA binding activity of other factors is highly diverse but is a hallmark of gene regulation. VDR DNA binding is facilitated by the retinoid X receptor (RXR) which acts as a heterodimeric partner (7, 8). *De novo* sequence analysis across many VDR sites confirms the presence of at least one and often multiple regulatory DNA sequence motifs, each comprised of a typical vitamin D response element (VDRE) of two hexameric DNA half-sites separated by three base pairs. This DNA structural organization represents a typical VDRE now observed at hundreds of genes and in all species examined. Interestingly, however, VDR binding at sites known to function in repression are more diverse, and often contain novel DNA motifs, suggesting that the VDR may be bound to these sites indirectly via an association with other DNA or non-DNA binding proteins serving additional regulatory functions. These observations at genome-wide levels both confirm and extend our understanding of several key features of vitamin D action via the VDR at target genes and are generally consistent with features of other transcription factors of this type. Perhaps the most important discovery arising from unbiased genome-wide analyses of not only VDR/RXR binding sites, but other transcription factor regulatory sites termed enhancers is the observation that these regulatory sequences are not located exclusively near target gene promoters as previously suspected. Rather, they can be found at sites that are commonly distributed within intergenic regions both upstream and downstream of the genes they regulate, often with unregulated genes interspersed, and frequently within the introns of the target gene itself or in unregulated genes located adjacent to the regulated gene or both (9–14). These sites can be located 10’s if not 100’s of kilobases distal to a gene’s promoter, the locations of which may have an impact on their function. Perhaps even more interestingly, this regulatory capacity is frequently comprised of multiple interacting components, often 2 to 10 or more of these functional regulatory enhancer regions. Finally, each of these individual components is frequently modular in nature, capable of binding multiple interacting factors, that can span a kilobase or less to more complex segments that can span many kilobases in size; super-enhancers and other large regulatory regions to which the VDR often binds appear to represent consolidated collections of multiple enhancers that interact with numerous regulatory proteins and extend to over 20 kilobases.

**Table 1:** Overarching principles of vitamin D action in target cells.

**VDR Binding Sites (The Cistrome): 2000-8000 1,25(OH)_2_D_3_-sensitive binding sites/genome whose number and location are determined by cell-type**
**Active Transcription Unit: The VDR/RXR heterodimer**
**Distal Binding Site Locations: Dispersed in *cis*-regulatory modules (CRMs or enhancers) across the genome; located in a cell-type specific manner near promoters, but predominantly within introns and distal intergenic regions; frequently located in clusters of elements**
**VDR/RXR Binding Site Sequence (VDRE): Induction mediated by classic hexameric half-sites (AGGTCA) separated by 3 base pairs; Repression mediated by divergent sites**
**Mode of DNA Binding: Predominantly, but not exclusively, 1,25(OH)_2_D_3_-dependent**
**Modular Features: CRMs contain binding sites for multiple transcription factors that facilitate either independent or synergistic interaction**
**Epigenetic CRM Signatures: Defined by the dynamically regulated post-translational histone H3 and H4 modifications**
**VDR Cistromes are highly dynamic: Cistromes change during cell differentiation, maturation, and disease activation and thus have consequential effects on gene expression**

Since multiple enhancers may control a single gene and the localization of transcription factors at sites on the genome do not identify the gene or genes they regulate, enhancer locations near a gene cannot be used to identify an enhancer’s target gene. Accordingly, the genome-wide number of transcription factor binding sites in a cell, for example, cannot be used to determine the number or identity of genes that are regulated by a given regulatory component. It would also appear that although the activation of some induced genes is temporally consistent with the direct regulatory actions of a transcription factor, other examples occur where this activity is not direct, but rather occurs following activation of cellular components that bind in a secondary fashion to a particular target gene. In the case of vitamin D, we will describe the regulation of FGF23 by 1,25(OH)_2_D_3_ and its receptor which may represent such an example. Mechanistically, it is also worth noting that although linear arrangements of binding sites at a gene locus are often schematically displayed, the actual arrangement *in vivo* is almost certainly a 3D complex of all or subsets or these regulatory units via looping. In addition, active enhancer regions near target genes that are characterized by open epigenetic chromatin structures provide access for regulatory factors that control the transcriptional output of the gene itself. This complexity also provides one example of why earlier studies using cell lines transfected with biased gene promoter plasmid constructs has frequently resulted in erroneous conclusions. These features will be observed in our studies of the renal genes for *Cyp27b1* and Cyp24*a1* and the genes for parathyroid gland produced PTH and skeletal produced FGF23.

## Current advances in transcriptional regulation *in vivo*

The technological advances as above have led to new methods to characterize the details of gene regulation both *in vitro* and particularly *in vivo*, the latter at molecular levels equivalent to those heretofore achievable only in cultured cells. Details now extend to the coupling of changing epigenetic architecture in response to genetic and epigenetic action at regulatory loci to the recruitment of additional transcription factors that play direct roles in promoting or inhibiting transcriptional output (3). These include detection of epigenetic readers that affect the distribution and release of RNA polymerase II across the transcription unit to those that influence the downstream mechanisms essential for transcription of the gene per se that results in the production of RNA transcripts. Of additional advantage is the fact that *in vivo* studies can frequently benefit from an assessment of the overarching functional roles of specific enhancers using CRISPR/Cas9 methods to induce mutations into the genome of both cells in culture and in animals *in vivo*. While many of the methods to be discussed herein were developed initially in cell lines, much of what we will be discussed below regarding the regulation of the genes involving 1,25(OH)_2_D_3_ production by the kidney and FGF23 production in bone were conducted in wildtype and mutant mice *in vivo*.

## Control of renal *CYP27B1* AND *CYP24A1* expression by PTH - raising the levels of endocrine 1,25(OH)_2_D_3_

### Induction of *Cyp27b1* expression by PTH in the kidney

We focus first on the regulation of 1,25(OH)_2_D_3_ production by the kidney. This hormone plays a central role in the regulation of mineral metabolism by virtue of its unique function to induce Ca and to a lesser extent P absorption from the diet via the intestinal enterocyte (15). Although this hormone also acts in bone and kidney as well, 1,25(OH)_2_D_3_ action in the intestine is novel. The primary regulator of 1,25(OH)_2_D_3_ production in the kidney is PTH which induces the expression of the *Cyp27b1* gene whose enzymatic product 25(OH)-1α-vitamin D_3_ hydroxylase (CYP27B1) is responsible for the synthesis of 1,25(OH)_2_D_3_ (16). As important, however, is the ability of PTH to reciprocally suppress the expression of *Cyp24a1*, whose product 1,25(OH)_2_-24-vitamin D_3_ hydroxylase (CYP24A1) is responsible for the initial degradation of 1,25(OH)_2_D_3_ (17). Accordingly, *Cyp24a1* is suppressed by PTH thereby reducing the degradation rate of 1,25(OH)_2_D_3_ and raising the levels of 1,25(OH)_2_D_3_. That the regulation of *Cyp24a1* is transcriptional in nature has only recently been defined in molecular terms, as will be discussed herein. Together, these two enzymes regulate the level of 1,25(OH)_2_D_3_, which is secreted into the blood where it functions as an endocrine hormone. Thus, the coordinated regulation of both *Cyp27b1* and *Cyp24a1* expression in the kidney by PTH is essential to both the production as well as the maintenance of appropriate levels of circulating hormonal 1,25(OH)_2_D_3_. Since the elaboration of PTH from the parathyroid glands is controlled by decreasing blood levels of Ca, an inverse relationship emerges between blood Ca and PTH, and thus control of circulating 1,25(OH)_2_D_3_ (18). As will be discussed in the next section, *Cyp27b1* is suppressed by the reciprocal actions of FGF23 and 1,25(OH)_2_D_3_, while *Cyp24a1* is coordinately induced by each of these hormones.

**Figure 2:**
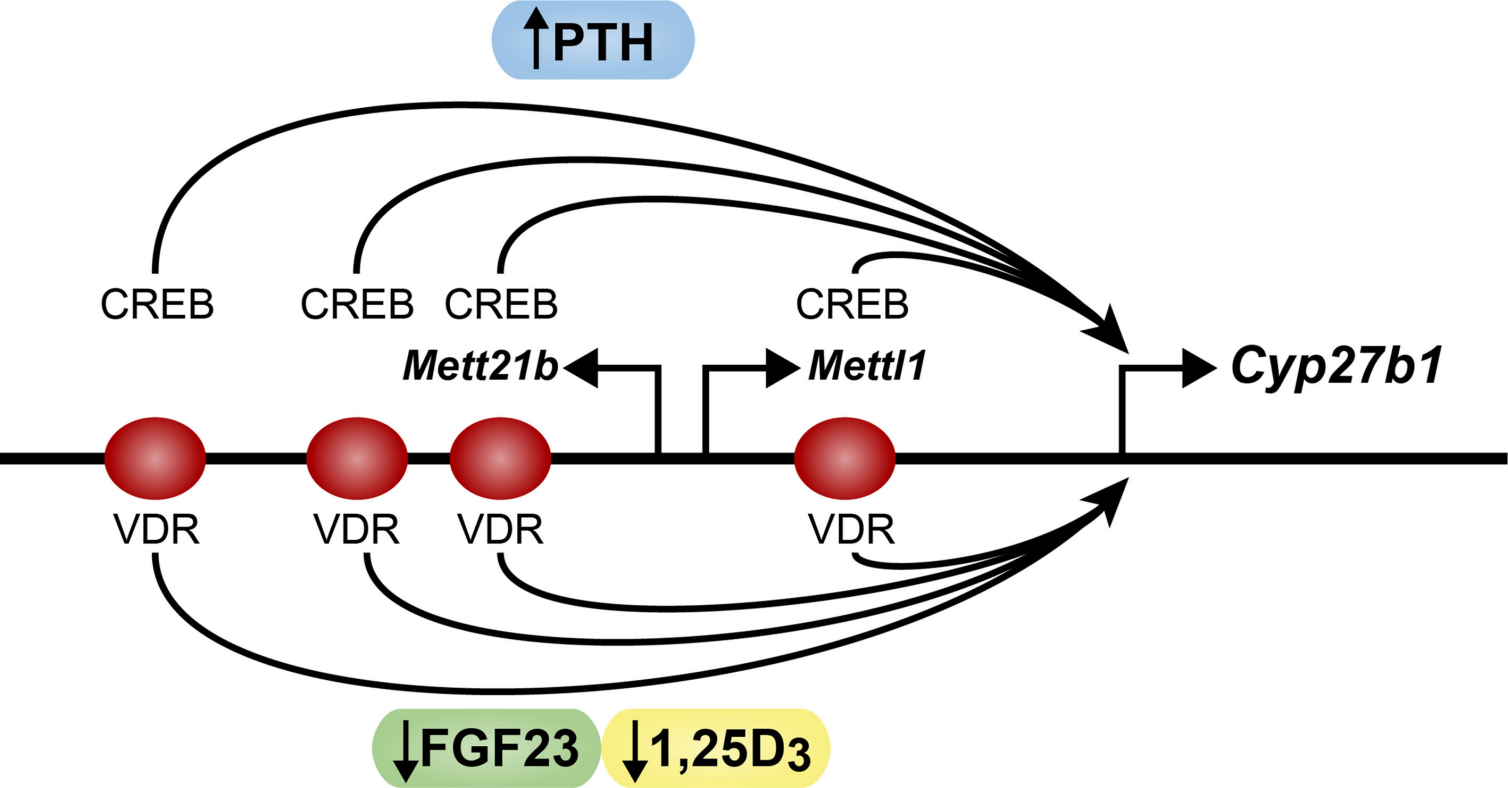
Schematic diagram of the mouse *Cyp2b1* gene. The *Cyp27b1*, *Mettl1* and *Mettl21b* genes are indicated. PTH (blue) sites of action at CREB are noted. FGF23 (green) and 1,25D_3_ (yellow) sites of action at the VDR are noted. Arrow indicates direction of transcription.

While studies several decades ago revealed the essential role for PTH in the control of renal production of 1,25(OH)_2_D_3_, it has been only recently that the mechanisms associated with the induction of *Cyp27b1* and suppression of *Cyp24a1* by this hormone have emerged. These findings were facilitated by the technics discussed above, including both ChIP-seq analysis and CRISPR-Cas9 mediated gene editing. To further the relevance of their application as well as our results, we applied these techniques to mice and conducted each of these analyses *in vivo*. As PTH is known to stimulate cAMP, we explored using ChIP-seq analysis the possibility that PTH might induce CREB at specific sites across the *Cyp27b1* locus in tissue derived from the kidney cortex of normal wildtype mice (19). Following *in vivo* treatment with exogenous PTH, we used antibodies to phosphorylated S133 CREB (pCREB) and discovered that pCREB could be detected at several sites upstream of the *Cyp27b1* gene, one located within an intron of the adjacent *Mettl1* gene and three located further upstream in an intron of the *Mettl21b (Eef1akmt3)* gene (**Figure 2**). None were found near the *Cyp27b1* gene promoter region nor immediately upstream of the promoter in an intergenic region that had been the focus of efforts by many others. While pCREB levels were not induced initially by PTH at these sites, later time course experiments revealed that the actions of PTH were more rapid than anticipated, resulting in a striking and transient upregulation of pCREB followed by a return to baseline as tested in the initial experiment (20). PTH also exerted a profound increase in histone acetylation (H3K9ac and H3K27ac) across each of these pCREB-containing sites even at earliest times, suggesting that PTH likely exerted an increase in DNA accessibility around these factor-containing sites. Each of these activities with PTH, however, preceded an increase in the production of PTH-induced *Cyp27b1* RNA transcripts. Most importantly, deletion of the pCREB containing site in the *Mettl1* intron (M1) resulted in a profound mouse phenotype insensitive to exogenous PTH and consistent with a previously prepared mouse containing a mutation in *Cyp27b1* that prevented the global expression of the active CYP27B1 enzyme (19, 21). The latter resulted in a total absence of circulating 1,25(OH)_2_D_3_ in these mice. Additional phenotypic features in both this unique *Cyp27b1* mutant mouse and the M1 deleted mouse included hypocalcemia, hypophosphatemia, and striking hypocalcemic bone disease. Deletion of the three collective sites in the *Mettl21b* intron (M21) in the genome of mice resulted in a much more modest phenotype comprised of only slightly reduced sensitivity to PTH, and normal levels of Ca, P, and 1,25(OH)_2_D_3_ (19). These and additional studies, together with the profound epigenetic observations induced by PTH, suggest that each of these pCREB sites mediate PTH action, but that the pCREB site in M1 is likely a dominant requirement for physiologically impaired PTH action (20). Importantly, a double mutant containing deletions of the pCREB site in M1 and the multiple sites in M21 resulted in a decreased basal level, a profound resistance to PTH, strikingly reduced circulating levels of 1,25(OH)_2_D_3_ and an extreme phenotype similar to that of the *Cyp27b1* null mouse; it was also slightly more severe than that seen in the M1 enhancer deleted mouse (21). Perhaps as interesting, a genomic analysis using ENCODE-produced DNase hypersensitivity (DHS) experiment revealed that all sites containing PTH-regulated pCREB exhibited an open chromatin profile present only in normal mouse kidney and absent in all other tissues examined (19, 22). More recent studies in isolated cells using ATAC-seq analysis revealed that this *Cyp27b1* enhancer profile could be seen only in renal proximal tubules and not in other renal cell types nor in a variety of other tissue cell types as well (2). Finally, that these enhancers were active was confirmed most recently through single cell RNA expression studies that revealed that *Cyp27b1* expression was limited to the renal proximal tubule segments S1 as well as S2 and S3 (23). While *Cyp27b1* was expressed at very low levels in many non-renal tissues (NRTCs), it was not responsive to PTH in any of these tissues (19). Thus, we conclude that the kidney module responsible for PTH mediated upregulation of *Cyp27b1* is unique to the kidney and responsible for the selective upregulation of *Cyp27b1* by PTH.

Our most recent studies using ChIP-seq analysis have revealed that while the levels of pCREB are not strongly induced by PTH, the mechanism through which the transcriptional activity of PKA induced pCREB is manifested is via the promoted recruitment of CBP, a CREB coactivator that exhibits histone H3K27 acetylation activity and is perhaps responsible for the ability of PTH to induce H3K27ac across the M1 and M21 sites (20, 23). Interestingly, additional chemical and genetic studies revealed that PTH via PKA also inhibits the salt-induced kinases (SIK) that result in the release of the cytoplasmic CREB regulated transcriptional coactivator (CRTC) class of CREB coactivators such as CRTC2 that also translocate to the nucleus where they bind to the CREB modules in M1 and M21 to facilitate *Cyp27b1* activation. Thus, activation of *Cyp27b1* in this case is mediated largely via the PTH mediated upregulation of two known coactivators of DNA-bound pCREB. The distinct role of each of these coactivators remains to be determined, however. Although widely expressed CBP retains multiple functions, one of which may be in the acetylation of histone H3K27; a second hypothetical function in the case of *Cyp27b1* may be to participate in the 3D repositioning of the four distal CREB enhancer sites at or near the *Cyp27b1* promoter (24). Perhaps as important in linking PTH to the upregulation of *Cyp27b1* is our observation that treatment with PTH results in the recruitment of BRD4 at each of the M1 and M21 pCREB sites (20). This histone H3 lysine acetylase reader participates in the initial phases of the recruitment of RNA polymerase II to *Cyp271b*. Accordingly, we observed that PTH also induced a striking increase in RNA polymerase II and an increase in enrichment of the epigenetic histone methylation mark H3K36me3 across the *Cyp27b1* gene body. The appearance of both factors reflects an increase in *Cyp27b1* transcription and provides direct links to the PTH-induced *Cyp27b1* RNA transcripts that are elevated in response to PTH treatment.

### Suppression of *Cyp24a1* expression in the kidney by PTH coordinately reduces the degradation of 1,25(OH)_2_D_3_

**Figure 3:**
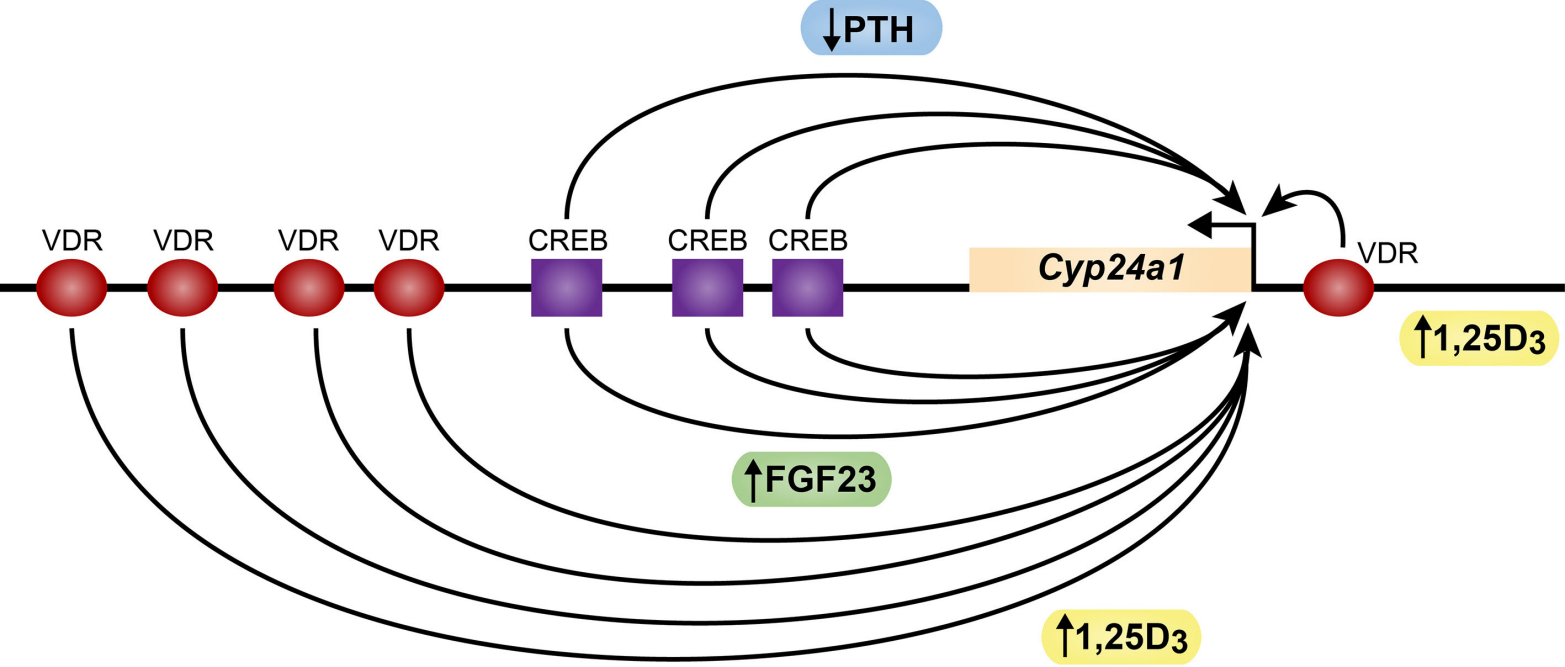
Schematic diagram of the mouse *Cyp24a1* gene. The *Cyp24a1* gene and downstream direction of transcription is noted. CREB sites (purple squares) and VDR sites (red ovals) are indicated. PTH (blue) sites of action negative action at CREB are noted. FGF23 (green) and 1,25D_3_ (yellow) sites of positive action at the VDR are noted.

While PTH induces *Cyp27b1* expression in the kidney, this hormone also imposes a reciprocal negative action on renal *Cyp24a1* levels (19). This reduction in *Cyp24a1* expression leads to reduced degradation of 1,25(OH)_2_D_3_ thereby facilitating the overall upregulation of renal 1,25(OH)_2_D_3_ that raises the hormones levels in the blood. While it has long been known that PTH suppresses *Cyp24a1* production, the mechanism of this decrease has evaded discovery; indeed, studies during the past decade suggested a post-transcriptional mechanism involving *Cyp24a1* transcript stabilization (25, 26). Interestingly, our recent ChIP-seq analyses of the renal *Cyp24a1* gene revealed that PTH suppressed *Cyp24a1* expression via a transcriptional mechanism, and that this downregulation was mediated via a large DNA segment that contains at least three widely spaced pCREB sites that are located immediately downstream of the *Cyp24a1* gene body (**Figure 3**) (27). Like *Cyp27b1*, an open chromatin feature also characterized the DNA at each of these pCREB sites along this downstream segment, but only in the kidney proximal tubules (2). Importantly, however, deletion of this large DNA segment (DS1) containing these pCREB sites in the mouse using CRISPR/CAS9 gene editing revealed a loss of PTH mediated *Cyp24a1* suppression, indicating that the pCREB features of this segment likely participated in renal *Cyp24a1* downregulation. Mechanistically, however, recent ChIP-seq studies revealed only modest suppression of small amounts of residual pCREB together with an apparent blockade of CBP and CRTC2 binding at these pCREB sites as well as a site at the *Cyp24a1* promoter in response to PTH (20). These limited changes in coregulator levels likely lead to the accompanying suppression of residual histone H3K9ac and H3K27ac and the absence of BRD4 observed at these sites, and therefore an inability of this gene to recruit RNA polymerase II. The corresponding absence of the transcriptional mark H3K36me3 indicated a lack of transcription. These features support the idea that PTH reduces *Cyp24a1* expression in the kidney at the genetic level, consistent with our observed suppression of *Cyp24a*1 RNA transcripts, and does so by opposing the ability of the pCREB modules to interact with CREB coregulators essential for *Cyp24a1* expression. Further studies will be necessary to identify the specific sites of action of PTH at one or more of these pCREB modules and to delineate the potential involvement of additional factors that may play a role in this suppression. Interestingly, as with pCREB components in the *Cyp27b1* gene, the pCREB modules in the *Cyp24a1* gene are uniquely absent in non-renal cells (NRTCs), supporting the concept that PTH is only capable of downregulating *Cyp24a1* in the kidney (27). Thus, as with *Cyp27b1*, the *Cyp24a1* gene retains regulatory features unique to the kidney. This reciprocal regulatory mechanism, together with the identified presence of both *Cyp27b1* and *Cyp24a1* in the same renal proximal tubules, support the coordinated involvement of both genes in the control of circulating 1,25(OH)_2_D_3_.

## Control of renal *CYP27B1* AND *CYP24A1* expression and regulation by FGF23 and 1,25(OH)_2_D_3_ - decreasing the levels of 1,25(OH)_2_D_3_

### FGF23 and 1,25(OH)_2_D_3_ decrease the expression of *Cyp27b1* and oppose PTH induction via pCREB sites at regulatory enhancers

Returning to our investigation of renal *Cyp27b1* regulation, we also explored the role of FGF23 and 1,25(OH)_2_D_3_ itself in renal *Cyp27b1* expression. Interestingly, while PTH speaks to the unique and direct upregulatory control of renal 1,25(OH)_2_D_3_ production in response to an increased need to elevate blood Ca levels, both FGF23 and 1,25(OH)_2_D_3_ itself are negative regulators that seek to downregulate the production of 1,25(OH)_2_D_3_ in response to unique physiologic/metabolic determinants (28–30). Despite this, while both suppress *Cyp27b1* expression, they may do so for different reasons, and as will be seen, by different mechanisms. Importantly, this suppression of *Cyp27b1*is also potentiated by the ability of FGF23 and 1,25(OH)_2_D_3_ to reciprocally induce *Cyp24a1*, thus collectively reducing blood 1,25(OH)_2_D_3_, as discussed in the next section (31). It is also worth noting that there is likely a distinction in the nature of the two signals in that while FGF23 represent a distal feedback mechanism prompted by circulating levels of P, 1,25(OH)_2_D_3_ and perhaps other regulatory components in the blood that control FGF23 expression from bone, identify a distal bone-kidney feedback axis. 1,25(OH)_2_D_3_ likely exerts its effects exclusively in the kidney via a short negative feedback mechanism. Nevertheless, despite an early understanding of the actions of 1,25(OH)_2_D_3_ and more recently of FGF23 to reduce 1,25(OH)_2_D_3_ production in the kidney via a downregulation of *Cyp27b1*, a delineation of the mechanisms involved have generally resisted research efforts. Importantly, while both FGF23 and 1,25(OH)_2_D_3_ suppress *Cyp27b1* expression in wildtype mice, deletion of either the M1 or M21enhancer regions using CRISPR/Cas9 gene editing *in vivo* reduced but did not eliminate the ability of either to suppress *Cyp27b1* expression (19). Deletion of both the M1 and M21 enhancers, on the other hand, fully abrogated the ability of both hormones to suppress renal *Cyp27b1* expression, suggesting that both hormones may act at similar sites (**Figure 2**) (21). Unfortunately, while we confirmed the presence of 1,25(OH)_2_D_3_-inducible VDR and RXR at each of these sites in M1 and M21 using ChIP-seq analysis, the lack of identification of a factor responsible for FGF23 suppression prevented unequivocal confirmation that these sites were the precise targets of FGF23. Moreover, since there were no striking differences between FGF23 and 1,25(OH)_2_D_3_ as measured by changes in residual pCREB abundance, or through striking suppression in the levels of residual CBP or CRTC2 at the pCREB sites within these M1 or M21enhancers, or in the ability of FGF23 to confer a differential profile of histone acetylation, these studies did not advance our understanding (20). Finally, as suppressors neither hormone was able to alter BRD4 binding at each of the pCREB sites, to influence residual levels of RNA polymerase II or to affect H3K36me3 levels across the gene body (32). We also observed that both FGF23 and 1,25(OH)_2_D_3_ oppose the upregulation of *Cyp27b1* in response to simultaneous treatment with PTH, although 1,25(OH)_2_D_3_ is more effective (20). These results suggest that renal *Cyp27b*1 is upregulated by PTH and that both FGF23 and 1,25(OH)_2_D_3_ appear to prevent both residual actions as well as the upregulation of *Cyp27b1* by PTH. These results provide novel mechanistic evidence for how these three hormones dynamically regulate the expression of renal *Cyp27b1* and therefore the production of circulating 1,25(OH)_2_D_3_.

### FGF23 and 1,25(OH)_2_D_3_ induce the expression of renal *Cyp24a1* and oppose PTH action via a dual FGF23/PTH sensitive pCREB containing region at the *Cyp24a1* gene promoter and at multiple sites downstream of the gene

*Cyp24a1* expression has long been known to be positively regulated by 1,25(OH)_2_D_3_ in tissues that include not only the kidney but in most VDR containing NRTCs as well. In contrast, while the more recent discovery that FGF23 regulates *Cyp24a1* has suggested that this may occur in several tissue types, the bulk of evidence suggests that FGF23 regulation of *Cyp24a1* is restricted to the kidney where FGF23 is biologically active (27). While FGF23 is known to mediate its extracellular actions via a coupling with Klotho that facilitates direct high affinity interaction with FGFR1C and FGFR3, the identity of the intracellular transcription factor and the mechanism whereby FGF23 induces *Cyp24a1* and certain other genes as stated earlier remain to be clarified. This may contrast with the suggestion that FGF23 activates the ERK1/ERK2 pathway that induces the transcription factor EGR1 (33–36). Importantly, however, the deletion of EGR1 in the mouse led to only a minor suppression in renal *Cyp27b1* expression while effects on *Cyp24a1* were not reported (35). And not surprisingly, many other immediate early genes are induced by numerous cellular regulators such as FGF23. Regardless, our efforts to identify the general genomic site(s) of action of FGF23 at the *Cyp24a1* gene suggest that FGF23 may act at open chromatin sites within the large DNA segment DS1 located downstream of the *Cyp24a1* gene that contains multiple pCREB binding sites. We speculate that the localization of FGF23 action in this region may facilitate in future studies the identification of the FGF23 transcription factor itself. On the other hand, 1,25(OH)_2_D_3_ has long been known to induce *Cyp24a1* expression via a specific couplet of VDREs located immediately upstream of the *Cyp24a1* promoter (37–40). These sites bind the VDR and its heterodimer partner RXR and appear to function in all tissues that express *Cyp24a1*. Our more recent studies using ChIP-seq analysis and novel cell-based and *in vivo* assays, however, have now revealed an additional large intergenic region located even further downstream of the *Cyp24a1* gene that contains numerous additional sites that bind the VDR/RXR heterodimer (**Figure 3**) (41). This discovery led to additional research that showed that many of these specific VDREs were functionally active in transfection assays. To functionally corroborate these earlier studies *in vivo*, we separately deleted the pCREB containing DS1 region downstream of *Cyp24a1* as well as the downstream region containing VDR binding sites (DS2) using CRISPR/Cas9 gene editing in the mouse genome and assessed the ability of FGF23 and 1,25(OH)_2_D_3_ to induce *Cyp24a1* following hormonal injection (27). Deletion of the DS1 segment resulted in the loss of FGF23 induction of *Cyp24a1* in the kidney, as seen similarly for PTH suppression, but resulted in only a modest effect on the ability of 1,25(OH)_2_D_3_ to induce *Cyp24a1.* Loss of the DS2 region also resulted in a modest decrease in 1,25(OH)_2_D_3_ induction in the kidney and other tissues as well. While these results may appear to conflict, it is worth noting that retention of the apparently dominant VDRE located near the promoter remained active *in vivo* and thus likely underlined the limited loss of 1,25(OH)_2_D_3_ response seen in the DS2-deleted mouse. This complexity suggests that additional experiments are needed to dissect the contributions of the promoter proximal VDREs in the *Cyp24a1* gene. However, we conclude that the sites of action of FGF23 and 1,25(OH)_2_D_3_ across this gene locus are likely distinct, with FGF23 sites located within the DS1 region and the 1,25(OH)_2_D_3_ inducible sites located within the DS2 region as well as at the promoter. Importantly, however, further analysis using ChIP-seq analysis revealed additional relevant information. Although both FGF23 and 1,25(OH)_2_D_3_ are strong agonists at the *Cyp24a1* gene, 1,25(OH)_2_D_3_ is a much more robust inducer of *Cyp24a1* than FGF23 (20). Thus, while each displayed common as well as distinct mechanistic effects on the gene, certain features appear to correlate directly with the more robust nature of 1,25(OH)_2_D_3_. For example, while neither affected the levels of pCREB present at the gene, both appeared to modestly induce CBP both across the downstream region as well as at the gene promoter, although 1,25(OH)_2_D_3_ exhibited more activity at DS2 and at the promoter proximal region. Interestingly, we noted that while both hormones exerted only nominal action on CRTC2, 1,25(OH)_2_D_3_ was much more effective at the *Cyp24a1* promoter. This surprising finding suggests that in addition to PTH, 1,25(OH)_2_D_3_ may also be able to influence CRTC2 migration to the nucleus. While the actions of FGF23 and 1,25(OH)_2_D_3_ are not entirely clear at the levels of pCREB downstream factors, the two hormones’ actions are largely similar at the level of epigenetic modification and the consequences that lead to RNA upregulation. In short, both hormones induce H3K27ac across similar regions of the gene, although 1,25(OH)_2_D_3_ is unique in that it also strongly induces H3K9ac. Finally, both hormones induce the recruitment of BRD4, although FGF23 is most active at DS1 whereas 1,25(OH)_2_D_3_ is most active at DS2, yet both are active at the promoter. Finally, RNA polymerase II is strongly recruited to the *Cyp24a1* gene under both hormone treatments, and this is reflected in the appearance of H3K36me3 across the gene body itself. Thus, there are both common as well as unique properties of both hormones, although it is clear 1,25(OH)_2_D_3_ is much more active at the *Cyp24a1* gene than FGF23, a feature that can be seen in the relative ability of the two hormones to induce *Cyp24a1* RNA transcripts. These data provide significant insight into the mechanisms through which FGF23 and 1,25(OH)_2_D_3_ regulate *Cyp24a1*, although additional work will be required to place the two hormones in perspective, not the least of which will be the identification of the transcription factor that mediates FGF23 action.

### Summary of the regulation of 1,25(OH)_2_D_3_ levels in the blood by PTH, FGF23 and 1,25(OH)_2_D_3_

**Figure 4:**
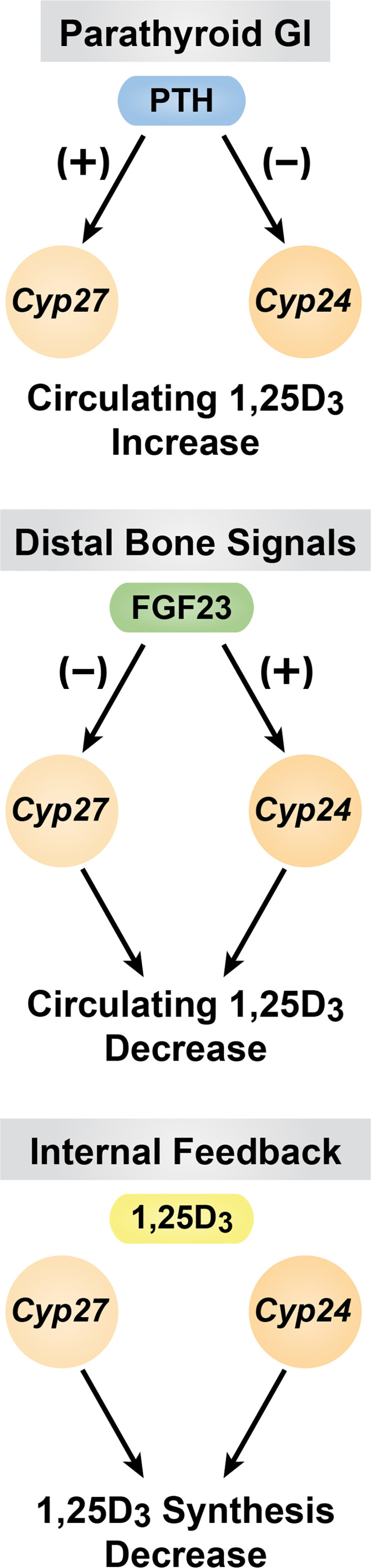
Summary of the of PTH, FGF23 and 1,25(OH)_2_D_3_ are diagrammed.

The above discussion defines the reciprocal co-regulation of *Cyp27b1* and *Cyp24a1* expression in the kidney that is central to the production and maintenance of endocrine 1,25(OH)_2_D_3_ by PTH, FGF23 and 1,25(OH)_2_D_3_ itself. These events are summarized in **Figure 4**. This importance is stressed at the *in vivo* level in mice when the M1 and M21 enhancers are deleted in the mouse, preventing the production of 1,25(OH)_2_D_3_ from being synthesized by CYP27B1. Under these conditions, the circulating levels of 1,25(OH)_2_D_3_ are strikingly reduced, but not eliminated. This level is insufficient to maintain the skeleton due to high PTH levels and reveals a striking *Cyp27b1* null-like phenotype (21). The residual blood levels of 1,25(OH)_2_D_3_ are due, however, to the substantial co-reduction in *Cyp24a1* expression that permits the small amounts of 1,25(OH)_2_D_3_ synthesized in the kidney to be sustained. We ultimately provide evidence of this hypothesis by rescuing this M1/M21-deleted mouse with a diet rich in Ca and P, which brings down PTH and raises FGF23 to normal levels thereby increasing renal *Cyp24a1* expression. Under these conditions, the levels of endocrine 1,25(OH)_2_D_3_ in the blood are reduced to undetectable concentrations, indicating the importance of the regulation of both renal *Cyp27b1* and *Cyp24a1* levels in the maintenance of circulating 1,25(OH)_2_D_3_.

## Control of PTH gene expression by 1,25(OH)_2_D_3_ and FGF23

As PTH represents the primary stimulator of the 1,25(OH)_2_D_3_ synthesis by renal *Cyp27b1* and likewise retards the hormone’s degradation via suppression of *Cyp24a1*, it should not be surprising that circulating 1,25(OH)_2_D_3_ might be a primary feedback suppressor of PTH expression. In fact, early studies suggested that was indeed the case. Accordingly, both cells in culture and rodents treated with exogenous 1,25(OH)_2_D_3_ exhibited a striking downregulation at the level of PTH mRNA in response to the hormone. Response was uncharacteristically slow in both, however, requiring 48 hours to achieve baseline levels suggesting additional mechanisms (42–44). Many of these mechanisms of PTH processing have been reported (45). One of the primary effects of 1,25(OH)_2_D_3_ and indeed most effective agonists of this hormone is to suppress PTH levels in various disease states that include chronic kidney disease (CKD) and perhaps other diseases characterized by inflammation (46–48). Zemplar represents one such analog of 1,25(OH)_2_D_3_ that has been effective in PTH suppression, although it is worth noting that this analog is considerably less effective in promoting calcium absorption than 1,25(OH)_2_D_3_ (49, 50).

**Figure 5:**
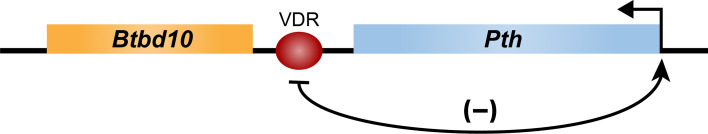
Schematic diagram of the mouse Pth gene located in blue. Arrow indicates direction of transcription. Location of a single VDR binding site (red oval) indicated at the 3’ end of the *Pth* gene, as assessed by ChIP-seq analysis of thyroparathyroid tissue.

The mechanism whereby 1,25(OH)_2_D_3_ might suppress PTH gene expression was explored several decades ago, when it was revealed that the VDR suppressed an artificial construct comprised of a reporter linked to the proximal region of the PTH gene. Accordingly, the VDR was found to bind to a sequence described as a negative VDRE located immediately upstream of the PTH start site (51). This mechanism was advanced based upon the use of traditional cell-based transfection assays of the era. Presently, the validity of this approach has been called into question, as it fails in general to account for numerous features of gene expression which were identified earlier in this manuscript. This result also remains in question as the ensuing years have not been able to substantiate the concept of a negative VDRE sequence. Indeed, the ability of hormones to suppress gene expression is quite complex, and generally involves the interaction of a suppressor with additional elements within an enhancer. Interestingly, recent studies focusing on the PTH gene using ChIP-seq analysis of thyroparathyroid tissue revealed that 1,25(OH)_2_D_3_ may induce VDR binding to a site located immediately downstream of the PTH gene (**Figure 5**). While preliminary, these data do not confirm VDR binding activity at a site upstream of the PTH gene.

Perhaps most interesting are recent studies in which a mouse model was examined wherein the VDR was selectively deleted from the parathyroid glands using the PTH gene promoter driving CRE (52). Surprisingly, loss VDR expression in the parathyroid glands had no effect on basal levels of PTH in these mice that could account for changes in blood PTH levels. There was also no effect of VDR deletion on the degree of parathyroid hypertrophy. These studies suggest that the effects of 1,25(OH)_2_D_3_ on PTH may be secondary to a hormone induced rise in blood Ca which likely reduces PTH levels.

Over the past decades, the discovery of the G protein-based calcium sensing receptor that modulates PTH regulation via Ca sensitivity has been the focus of research on PTH regulation (53). The literature on this subject is vast and has led to the development of therapeutically relevant compounds that are in use currently.

### Regulation of PTH by FGF23

The literature is replete with studies suggesting that FGF23 regulates PTH. These studies support both positive as well as negative effects and are almost always conditional (54). Given that lack of insight into the impact of environment on FGF23 action on PTH, a definitive impact remains to be established mechanistically.

### Summary of PTH gene regulation

This short summary outlines what is largely known historically of PTH gene regulation. It is not surprising that key details remain to be explored, as useful parathyroid cells are generally not available thereby restricting the use of current techniques described earlier in this manuscript.

## Induction of *Fgf23* gene expression in bone by PTH and 1,25(OH)_2_D_3_

FGF23 represents a long sought after bone-derived peptide hormone that regulates P homeostasis. FGF23 binds to the fibroblast growth factor receptors (FGFRs) FGFR1c and FGFR3 in the kidney, decreasing P reabsorption in the proximal tubules via the positional regulation in the brush border of Na,-P transporters NAPI2A and NAPI2C (29, 30, 55). This repositioning results in the loss of P recovery by the kidney and ensuing hypophosphatemia. FGF23 also downregulates renal *Cyp27b1* and induces *Cyp24a1* gene expression as detailed earlier, thereby reducing the synthesis of 1,25(OH)_2_D_3_ and thus the potential intestinal uptake of both Ca and P. The mechanism of action of FGF23 is dependent upon the exceptionally high affinity of this hormone for the renal FGF receptors by virtue of their interaction with α-klotho, a renal co-receptor that functions to elevate the subsequent binding affinity of FGF23 for the appropriate FGFRs (33). Activation of the FGFRs is believed to result in a triggering of the ERK1/ERK2 signaling pathway that is known to activate the transcription factors EGR1 and perhaps other immediate early genes. While these pathways are known to be involved in the regulation of certain genes by FGF23, the loss of EGR1 via mutation in mice, as stated earlier, had little to no effect on *Cyp27b1* expression, suggesting that other unidentified transcription factors may be targets of this pathway (35).

FGF23 is regulated by a myriad of physiologic modulators that represent not only transcriptional inducers, but factors that are involved in FGF23 processing as well. This complexity emerged as a result of a number of genetic P related disease states in humans that not only facilitated the discovery of FGF23 but identified many of the key features of additional components essential to P related control and post transcriptional regulation of FGF23 as well. Significant interest has been sustained over several years now in understanding the transcriptional regulation of *Fgf23* by not only the primary mineral regulating hormones such as PTH and 1,25(OH)_2_D_3_, but factors that participate in this regulation of *Fgf23* at the genomic level. Indeed, early studies suggested the locations of VDRE-like sequences within the upstream region of the *Fgf23* gene using traditional transfection assays (56–58). These were suggested to mediate a secondary activity of the VDR/RXR heterodimer; others suggested that the actions of PTH action occurred via the NURR1 transcription factor (59). Perhaps as important as the above hormones, however, is role and mechanism of action of P itself. Due to the temporal features of virtually all dietary P studies, it remained unclear whether P action at the *Fgf23* gene was transcriptional or post-transcriptional in nature (60). In some cases, factors involved in the transcriptional regulation of *Fgf23* by immune activators, iron and other inducers have been observed, given previous knowledge of their pathways. In view of some of the uncertainties that have emerged, we took an exclusively *in vivo* approach in which to identify the sites of action of PTH, 1,25(OH)_2_D_3_ and P at the *Fgf23* gene, with the supposition that the identification of the precise genomic regions that mediate such regulation might provide clues as to the nature of transcription factors involved. While certain factors seem likely to participate in the induction of *Fgf23* by PTH and 1,25(OH)_2_D_3_, for example CREB and the VDR, there do not appear to be likely candidates for a pathway(s) that might mediate P regulation, necessitating an unbiased search. As will be seen in the following description of our current efforts, the presumption of involvement and the mechanisms of such involvement of anticipated specific targets in these early studies has been highly misleading. Indeed, for example, while it is known that the VDR mediates the actions of 1,25(OH)_2_D_3_ at numerous target genes, it appears that the pathway whereby *Fgf23* is induced by 1,25(OH)_2_D_3_ may be indirect and involve the activity of secondary factors. CREB activation represents a natural mediator of PTH. However, although many of the upstream components of PKA induction by PTH are now well known, the molecular mechanisms through which this pathway triggers controls of SIK inhibition and regulates CREB activity remain to be clarified, as highlighted in our discussion of PTH regulation of coregulators of CREB at the *Cyp27b1* gene (20, 23). Furthermore, PTH activates additional signaling pathways and many of the factors that mediate gene regulation via these pathways also remain unclear as well. P on the other hand, requires first the initial determination of whether a transcriptional mechanism of action may be involved prior to the search for mediators, including not only the pathways and the transcription factors involved but the identity and potential role for cellular P sensitizers analogous to that for Ca in the parathyroid glands as well (61–64). Accordingly, we approached our search in an unbiased fashion using several of the techniques outlined in the earlier section on gene regulation, together with additional methods that have been mostly *in vivo* in the mouse.

### PTH upregulates *Fgf23* expression in bone *in vivo via* a potential CREB factor

**Figure 6:**
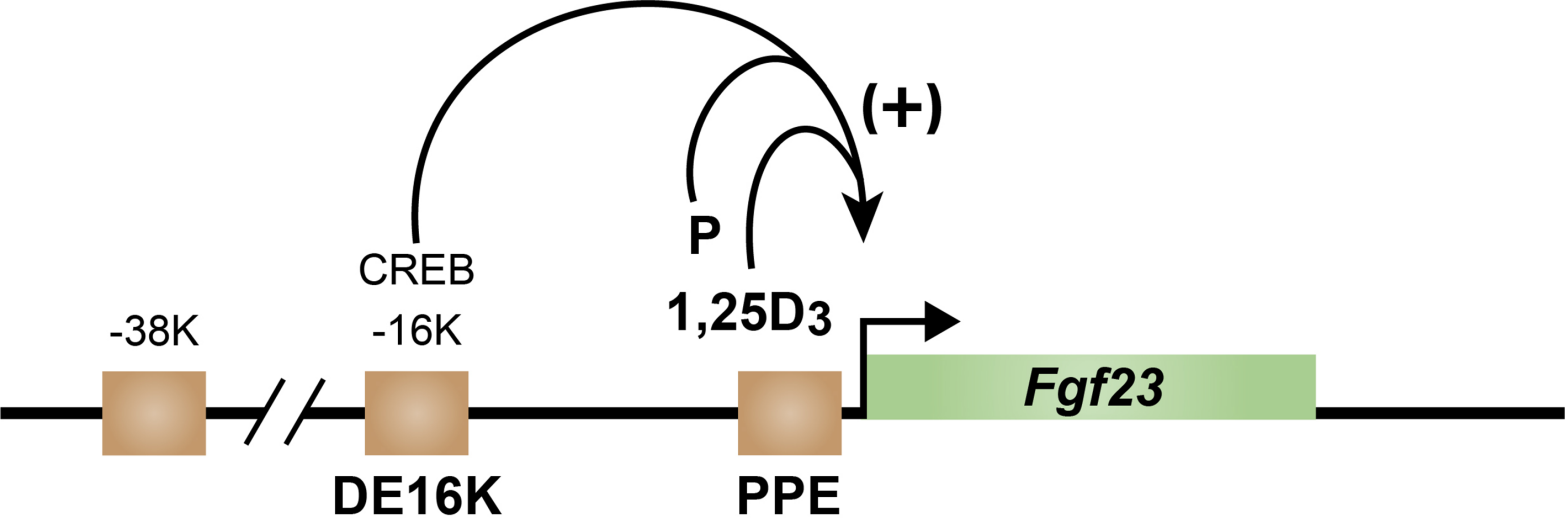
Schematic diagram of the mouse *fgf23* gene located in green with arrow indicting direction of transcription. Brown squares indicate regions of increased epigenetic acetylation. Positive regulation by PTH occurs from the CREB site in the DE16K enhancer (DE16K) and by 1,25D_3_ and P at the promoter-proximal enhancer (PPE).

Early exploration of the *Fgf23* gene using ChIP-seq analysis revealed four regions across the *Fgf23* gene locus in osteocytes that were enriched for H3K27ac, an epigenetic mark that has been associated with an open chromatin state critical for enhancer function (**Figure 6**) (65). Two sites were located upstream of *Fgf23* gene at -16 kb and -38 kb, one near the promoter region, and one in a downstream intron at +7kb. This discovery prompted the CRISPR mediated deletion of each region and a search for loss of functional activation by PTH. While PTH induction of *Fgf23* mRNA in bone was lost in the distal -16kb (DE16K) deleted mouse, no effect was seen in any of the other mouse mutants. Further ChIP-seq analysis of osteocytes revealed the presence of pCREB exclusively at this distal enhancer. Interestingly, recent studies suggest that several SIK inhibitors that promote the recruitment of the CREB receptor transcriptional coactivator 2, (CRTC2) to the *Cyp27b1* gene in mouse kidney also induces *Fgf23* in the bones of those same mice (20). These results suggest that the mediator of PTH action may indeed be pCREB seen bound at DE16K. While further work will be necessary to establish the precise role for pCREB at the *Fgf23* gene, its location 16kb upstream does not contain a NURR1 site. Additional ChIP-seq analyses also fail to identify the presence of NURR1 across the entire *Fgf23* binding motif while revealing binding at other additional known sites for NURR1 including the osteocalcin gene in response to PTH. Further studies of NURR1 activation by PTH and 1,25(OH)_2_D_3_ in rat UMR bone cells revealed that the pattern of temporal induction of NURR1, as well as NURR2 and NURR3 and other immediate early genes such as *EGR*1, 2 and 3 are also upregulated (Pike, unpublished)). These studies allow us to conclude that it is unlikely that the mediator of PTH action at *Fgf23* in bone is NURR1. In mice, the rapid actions of exogenous PTH resulted in PTH induction of *Fgf23* bone mRNA, and prevented the delayed but coordinated detection of upregulated iFGF23 in the blood, as will be seen for 1,25(OH)_2_D_3_ and P.

### 1,25(OH)_2_D_3_ upregulation of *Fgf23* expression in bone *in vivo* may involve a secondary process

1,25(OH)_2_D_3_ upregulates *Fgf23* expression at the transcriptional level and is known to require the presence of VDR in both mice and in cells in culture. In cells, this induction is rapid and reminiscent of most other genes that are induced by this hormone (57). Nevertheless, our earliest studies of *Fgf23* in the osteocyte revealed that despite this classic temporal regulation of *Fgf23* by 1,25(OH)_2_D_3_, we were unable to identify the presence of the VDR at any sites across the *Fgf23* gene locus, despite its presence at numerous known vitamin D target genes in these bone cells (66). Our most recent attempts to identify VDR as well as RXR binding sites at *Fgf23* using ChIP-seq analysis in other bone cell lines were also unable to identify the presence of these two receptors, again despite the presence of this heterodimer at all known genes we inspected. These data suggested that the induction of *Fgf23* by 1,25(OH)_2_D_3_ might be indirect, and mediated by the ability of this hormone and its receptor to rapidly induce one or more secondary transcription factors that might interact directly with the *Fgf23* gene. This result prompted further exploration of the *Fgf23* gene and revealed that deletion of only the promoter proximal (PPE) but not the DE16K region of *Fgf23* in mice using CRISPR/Cas9 gene editing was essential for loss of 1,25(OH)_2_D_3_ induction of *Fgf23* (67). The unusual nature of this activity of 1,25(OH)_2_D_3_ dictated a further unbiased dissection of the PPE by creating a series of mouse mutants containing unique genomic deletions upstream of the *Fgf23* start site. Numerous internal mutations and specific deletions were also created as well. These series of mutant mice were then explored for loss of function at the *Fgf23* gene as above to map in an unbiased manner response to not only 1,25(OH)_2_D_3_ but to P (see below) and other activators as well. Accompanying the quantification of bone *Fgf23* mRNA, we also determined whether any changes in the level of mRNA resulted in a change in the circulating levels of intact (i) FGF23 (iFGF23); a change in both was necessary for us to conclude that the deletion resulted in a loss of function. The loss of response to 1,25(OH)_2_D_3_ occurred in mutant mice in which deletions were created downstream of the VDRE-like sequence proposed by Liu et al. (58) and upstream of a site suggested to contain a proposed NURR1-like site termed the NURR1/VDRE like sequence as discussed above which was believed to be a potential target for PTH as well as 1,25(OH)_2_D_3_ (57, 59). Internal base mutations of this NURR1/VDRE sequence alone as well as small deletions both upstream and downstream of this site had no effect on the ability of 1,25(OH)_2_D_3_ to induce bone *Fgf23* mRNA or to upregulate blood levels of iFGF23. These data suggest that neither the upstream VDRE-like sequences proposed earlier nor that of the NURR1/VDRE sequence proposed more recently, nor sites more proximal to this latter proposed site mediates the actions of 1,25(OH)_2_D_3._ A recent alternative evaluation of this region appears to concur, speculating in detail that this site and transcription factors other than NURR1 may be involved (15). Perhaps more importantly, the DNA segment located between these two hypothetical mediators of 1,25(OH)_2_D_3_ does not contain a potential VDRE-like DNA motif capable of directly binding the VDR, supporting our earlier suggestion that the VDR does not bind directly within the *Fgf23* gene locus. Thus, we are currently exploring this mapped region of 1,25(OH)_2_D_3_ sensitive genomic space that appears to be functionally relevant *in vivo* in an unbiased search for potential transcription factor candidates that are amenable to technical confirmation as mediators of 1,25(OH)_2_D_3_ action. Importantly, these data suggest a novel, yet transcriptionally consistent, genomic mechanism whereby 1,25(OH)_2_D_3_ induces the expression of an important gene involved in mineral homeostasis.

### P upregulates *Fgf23* expression in bone *in vivo via* distinct acute and chronic mechanisms

P induces an upregulation of *Fgf23*, even in mice devoid of the VDR (31). The mechanism by which this mineral functions to induce *Fgf23* as well as other genes is not known. This appears to be largely because the study of this mineral over many decades *in vivo* has involved dietary P loading experiments of a generally prolonged nature, wherein lower or elevated P levels in the diet were delivered to mice. This delivery system results in a lengthy delay (days) in the measurement of P response, including the upregulation of *Fgf23* and prevented exploration of mechanisms whereby P might induce *Fgf23*. To overcome this issue, we developed a method whereby elevated dietary P could be rapidly delivered to mice and the upregulation of *Fgf23* mRNA evaluated within several hours as described previously; importantly, both the upregulation of *Fgf23* mRNA as well as blood iFGF23 could be detected within that time frame (60). Subsequent studies revealed an elevated response to high P as early as 1 h. The rapidity of this response suggested that the mechanisms might be transcriptional in nature. More definitively, however, we also discovered that deletion of the PPE but not the DE16K of the *FGF23* gene resulted in a loss of this rapid induction by high P, providing strong support for the idea that this mechanism likely involved transcriptional regulation. Surprisingly, however, despite this loss of acute induction, P appeared to upregulate *Fgf23* mRNA and iFGF23 through an additional chronic mechanism apparent in this PPE mouse mutant beginning within 24 h that resulted in normal levels of *Fgf23* mRNA and blood iFGF23 by 48 h. It is worth noting here that these results did not appear to arise through an unusual underlying feeding mechanism inherent to the approach, as diet without P was used as feeding controls. Thus, high P appears to induce *Fgf23* by two different processes that differ temporally and mechanistically.

To further explore the site of acute action of P at the *Fgf23* gene, we mapped the loss of P induced response of *Fgf23* mRNA and iFGF23 in each of the mutant mice developed for our studies described above with 1,25(OH)_2_D_3_. As with the steroid hormone, a loss of P response was identified both upstream of the NURR1/VDRE-like site. Deletion of the NURR1/VDR-like site also had no effect on loss of *Fgf23* mRNA or *Fgf23* induction. Thus, a single upstream DNA segment identical to that for 1,25(OH)_2_D_3_ induction was apparent (67). It is worth noting, however, that this segment was relatively lengthy. In addition, the time course for P was much more rapid for P than for 1,25(OH)_2_D_3_. Therefore, it cannot be concluded that both P and 1,25(OH)_2_D_3_ target the same DNA sequence(s) or utilize the same transcription factor(s). Studies are in progress with additional mutant mice to refine the sites of action of P at the *Fgf23* gene and to identify functional motifs useful for further exploration of the mechanism of acute P action.

In order to gain additional support for the identity of possible targets of both 1,25)OH)_2_D_3_ and P, we also explored the general response in bone via RNA-seq analysis Our working hypothesis is that a genome wide response as determined by an RNA-seq analysis and subsequent analysis of regulated RNAs might provide information regarding not only a relationship between the two inducers, but might reveal additional useful pathways for P in particular, and possible reveal novel transcription factor candidates as well. Of course, the actions of each of these inducers may not involve the induction of regulators via transcription but rather the activation of other signaling pathways as well. One pathway that is common to both 1,25(OH)_2_D_3_ and P is an upregulation of Wnt/β-catenin signaling, observed via an increase in the expression of numerous Wnts and the downregulation of numerous Wnt inhibitors (68). However, this overlap is not broadly displayed and many of the genes that are regulated by 1,25(OH)_2_D_3_ do not overlap those regulated by P. Future studies will be necessary to determine the relevance of these data with those derived from our mechanistic studies of *Fgf23* regulation in the mutant mice.

## Concluding remarks

The regulation of mineral metabolism is highly complex and involves three dominant hormones that includes PTH, 1,25(OH)_2_D_3_, and FGF23. While these hormones coordinate the regulation of both Ca and P in multiple organs including the intestine, bone, and kidney, they also exert additional hormone-dependent actions in additional tissues that are unrelated to the control of mineral metabolism. Relative to mineral metabolism, numerous diseases are associated with the actions of these hormones; altered mineral metabolism also emerges due to a wide variety of human disease states. Importantly, each of these hormones coordinate actions that include the regulation of the other two. While this has been widely known, the molecular mechanisms responsible for this inter-regulation are only now emerging, due in part to the development of new technologies that have enable the studies described herein.

## Author contributions

JP drafted and edited the manuscript; MM edited the manuscript; SL edited the manuscript. All authors contributed to the article and approved the submitted version.
